# A qualitative study into female sex workers’ experience of stigma in the health care setting in Hong Kong

**DOI:** 10.1186/s12939-019-1084-1

**Published:** 2019-11-14

**Authors:** Haixia Ma, Alice Yuen Loke

**Affiliations:** School of Nursing, The Hong Kong Polytechnic University, Hung Hom, Kowloon, GH 525 Hong Kong, China

**Keywords:** Female sex workers, Stigma, Coping, Identity, Accessing health services

## Abstract

**Background:**

Hong Kong has gained a good reputation for its quality public health care services. However, there is a growing recognition that social stigma is a potential obstacle when female sex workers (FSWs) access health care services. There are a lack of studies focusing on how FSWs experience and cope with stigma when accessing health care services in Hong Kong.

**Objective:**

This study aims to explore how FSWs experience stigma and develop coping strategies when accessing health care services in Hong Kong.

**Methods:**

This is a qualitative interview study. Staff of non-governmental organizations (NGOs) that serve sex workers in Hong Kong facilitated the process of recruiting the participants. In-depth individual interviews were conducted with 22 FSWs, focusing on their experiences of stigma and coping strategies when accessing health care services. A directed content analysis approach was adopted to analyze the data.

**Results:**

The interview data can be grouped into three themes: experience of stigma in the health care setting; coping with the stigma of sex work; and the call for non-judgmental holistic health care.

**Conclusion:**

This study contributes to an understanding of the experience of stigma and stigma coping strategies of FSWs when accessing health care services in Hong Kon*g. stigma* remains an important issue for a large proportion of FSWs when they seek timely professional help, openly disclose their sex work identity, and receive comprehensive health care services. The study also highlights the need to address multiple healthcare needs of FSWs beyond STDs. Moreover, the study contributes to increasing awareness of, and respect for, the human right of FSWs to receive non-discriminatory health services.

## Introduction

According to the classic definition of stigma provided by Erving Goffman (1963), stigma is “an undesirable or discrediting attribute that an individual possesses, thus reducing that individual’s status in the eyes of society.” (p.3) [1]. Stigma is a process by which the member of the stigmatized group holds a devalued identity. Female sex workers (FSWs) are stigmatized and marginalized around the world. They are generally not accepted in society and are regarded as criminals, immoral troublemakers, sexual deviants, and vectors or reservoirs of disease [2, 3]. Criminalization of sex work could further entrench the social stigma and increase FSWs’ vulnerability to violence [4].

The conceptualization of stigma and the extensive literature on the stigma of sex workers suggested that FSWs may experience multiple levels of stigma, namely social stigma, structural stigma, and self-stigma [5–7]. Social stigma is the attitudes and beliefs that the general public hold toward the stigmatized population [5]. Structural stigma refers to the ways institutions legitimize and perpetuate stigma [6]. For example, health care providers’ stereotypes about FSWs may result in refusal of treatment, sub-optimal care, humiliation, breach of confidentiality and privacy, and other forms of discrimination [8, 9].

At the individual level, self-stigma refers to the stigmatized members may experience, anticipate, and internalize the social stigma and the structural stigma [5]. The experienced stigma refers to the actual occurrence of prejudice and discrimination experienced by the member of the stigmatized groups. The anticipated stigma refers to the expectations that the members of the stigmatized group may experience stigma if their stigmatized condition has been revealed [10]. The internalized stigma arises when the individuals who belong to a stigmatized group accept and internalize the society’s negative attitudes [5]. FSWs may anticipate or experience negative attitudes or rejection at the health care setting and in the community. Those who suffer from self-stigma may have low self-esteem and avoid seeking help [11, 12]. To avoid stigma and discrimination in the health care setting, FSWs are more likely than the general population to hide private information and to set boundaries with health care providers when they need to disclose their occupation [11, 12].

Hong Kong Special Administrative Region of the People’s Republic of China, is a city with approximately 7.5 million people [13]. It has been recognized as a leading global financial centre. Also, Hong Kong has a good reputation for its quality, efficiency, accessible, and affordable public health care services. The hospital authority provides public hospitals and related medical services. The Department of Health focuses on disease prevention and health promotion [14]. The social hygiene clinics under the Department of Health provide free services for skin diseases and sexually transmitted diseases (STDs) for its citizens [15]. Besides, a number of local non-governmental organizations (NGOs), such as the Action for Reach Out, Ziteng, JJJ Association, also offer free and confidential STDs services and/or gynecology examinations for FSWs. Moreover, people could enjoy personalized services from the private sectors if they are willing to pay for higher health care costs.

The exact number of sex workers in Hong Kong is difficult to estimate since they often work in secrecy. A local NGO for sex workers estimated the number of sex workers might range from 20,000 to 100,000 in 2001 [16]. Since there has been a dramatic increase in the number of sex workers crossing the border from mainland China into Hong Kong [16–18], the current number of sex workers may exceed what was estimated around two decades ago. The act of prostitution itself for Hong Kong residents is not illegal, but it is illegal under certain circumstances according to the local law, such as controling sex workers for prostitution, soliciting for immoral purpose in a public area, or living off the earning of a sex worker [19].

Despite universal health coverage, comprehensive services available at public hospitals, and free sexual and reproductive health care services offered by social hygiene clinics and numerous NGOs in Hong Kong [15], many FSWs remain reluctant to seek timely treatment [20–23]. A survey among 89 FSWs in Hong Kong reported that 55.1% of participants had never taken STDs tests [20]. Another study among 293 FSWs reported that 43.2% of them used illegal clinics for induced abortion [22]. A more recent survey among 340 FSWs in 2013 has found that the prevalence of HIV and STD screening test in the previous year was 44.4 and 45.0%, respectively [23]. Besides, self-medication is not uncommon among FSWs. The prevalence of self-medication estimated from 494 FSWs who had suspected STD symptoms in the previous year was 14.1% [24].

The factors affecting the uptake of health care services by sex workers have been extensively studied. A synthesis of the relevant literature suggests that multiple barriers, including those at the intrapersonal, interpersonal, institutional, community, and policy levels, could hinder sex workers from accessing health care services. Social stigma has been recognized as a key barrier that is present at different levels [7].

Although the recognition of the impact of stigma on the access of health care by FSWs has grown, there are a lack of researches focusing on how FSWs experience stigma and cope with it when accessing health care services in Hong Kong. According to the social-cognitive model, the response to self-stigma could either diminish one’s self-esteem and self-efficacy or increase one’s sense of self-empowerment [25, 26]. Individuals who accept societal stigma as legitimate may suffer from low self-esteem. While an individual who perceived societal stigma as illegitimate may resist the stigmatized identify and bolster their empowerment [25, 26].

This study aims to fill this gap by including the voices of FSWs in an attempt to explore how they experience stigma and develop coping strategies when accessing health care services. It should be noted that this work is not merely focused on the illness related to sex work, but also include other health conditions that require access health care services. The findings of this study could raise awareness among health care professionals of the health risks and health care needs of sex workers, and the obstacles that they encounter when accessing health care services. This will make it possible to develop appropriate intervention programs to allow sex workers and other marginalized populations to receive equal access of health care services in an environment where they feel respected.

## Methods

A qualitative approach was adopted as this approach emphasized people’s lived experience and suited for health care research and stigma research [27, 28]. This approach would allow the researcher to get rich and in-depth information of FSWs stigma experience when accessing health care services. The individual interview was considered best suited to topics that are sensitive in nature and was employed in this study [29].

### Recruitment of participants and study setting

FSWs are a hard-to-reach population. Since local NGOs had established a relationship of trust with FSWs, the potential participants were recruited with the support of NGOs, including the Action for Reach Out (AFRO) and the JJJ Association. These organizations focus on the social inclusion of FSWs and assist them in dealing with health, safety, legal, and human rights issues. They also run outreach teams and are in regular contact with a number of FSWs throughout the city. One of the authors received training from NGOs and worked with them during outreach activities, campaigns, and events related to sex workers’ rights. The long-term relationship between the author and NGOs contributed to the success of recruitment.

After obtaining ethical approval for this study from the Human Subjects Ethics Sub-committee of the Hong Kong Polytechnic University (Reference Number: HSEARS20181122001), potential FSWs were recruited. Non-probability methods, such as convenience and snowball sampling, are often used to recruit hard-to-reach populations [30]. A combination of convenience and snowball sampling techniques were used to recruit FSWs in this study.

The criteria for inclusion in the study were FSWs who were: 1) over 18 years of age; 2) currently engaged in sex work, defined as having offered to perform at least one sexual service for money within the last 4 weeks; 3) able to speak Cantonese/Putonghua; and 4) able to give informed consent. Excluded from the study were: 1) those unable to speak Cantonese/Putonghua; 2) who had been diagnosed with and were currently undergoing medical or psychological treatment for a serious psychological health problem such as psychosis, bipolar disorder, and/or severe affective disorder; 3) who had self-reported current suicidal ideation and/or attempts; 4) who refused to give their informed consent to participate in the study.

A safe and comfortable environment was essential to ensure the safety for both FSWs and the interviewer. The interview took place at the office of one NGO or at one-woman brothels accompanied by an NGO staff member. Also, due to the sensitive nature of the topic, the “same-gender” interviewing was considered beneficial to build rapport between the researcher and the participants. All the interviews were conducted by a female research student who came from a postgraduate research background and had received qualitative interview training during her master of public health and doctor of philosophy programmes. The research student was a registered nurse, who had experience in providing sexual and reproductive health care services to FSWs at the local NGO, and had gained rich experience in talking with FSWs in a sensitive, open, and non-judgmental manner. Moreover, the research student had received Mental Health First Aid training in Hong Kong, and the skills that she had learnt from the course helped her to assess the mental health of the participants during the interview.

The researcher did not contact FSWs directly. Instead, staff of NGOs contacted potential participants directly via phone calls or during outreach activities, and provided them with the information sheet and the consent form of the study. They described the aims of the study, went through the information sheet, and invited FSWs to participate. Once the potential participant agreed to participate, staff of NGOs confirmed with the research student about the eligibility of the participants, the interview date, time, and venue by email or instant message (i.e., WhatsApp).

Before the interview started, the research student started the conversation casually to establish rapport with the participants. Then, the research student explained the aims of the project and checked potential participants for eligibility. She then invited those who were eligible to participate and obtained their informed consent prior to conducting the interview.

The author would disseminate the results to the participants upon their request. The participants were given options for receiving the research findings from journal papers, seminars, one-on-one meetings, and other social media (i.e. Facebook, Twitter, WeChat, or WhatsApp). Also, the two NGOs would be involved in the dissemination of the findings of the study to FSWs community and healthcare organizations.

### Data collection

The semi-structured interview was used, as it is considered as a flexible tool to capture the voice and experience of the participants. This method uses a prepared interview guide, but the open-end and probing questions are flexible to allow the participants to recount their experience and even expand the original questions and responses [27, 31].

The semi-structured interview guide was developed by the research student based on a review of the literature and on her previous experience with working and volunteering at an NGO that offers health services to FSWs. The proposed questions were further discussed with a university professor who is an expert in women’s health and a social worker from an NGO. The interview guide covered the following topics: FSWs’ health and service needs, access and experience with health care services, the attitude of health professionals, the disclosure of FSWs’ status in the health care setting, and whether they had any recommendations for better health care services (See Additional file 1 -Interview Guide). In the interviews, the interview questions were only used as a guide; the prompts were used to explore the participants’ concerns in depth.

From December 2018 to February 2019, semi-structured individual interviews were conducted with FSWs in Hong Kong. The number of the participants involved were determined by data saturation when no new data was being found from the participants [32]. The theoretical saturation was reached when 22 interviews had been completed. The interviews lasted from 42 to 124 min. The interview data were transcribed and briefly analyzed within 1 week after the holding of the interview.

All the participants provided written consent with pseudonyms. Most of the individual interviews were audio-recorded, although five participants refused to be recorded during their interviews, and handwritten notes were taken during those interviews. In addition, communication with a hearing and speech impaired FSW was conducted by writing notes on a computer. Field notes were written down to complement taudio-recordings.

### Data analysis

Directed content analysis is adopted when “the existing theory or prior research exists about a phenomenon that is incomplete or would benefit from further description” (p.1281) [33]. The present study started with the previously developed conceptualization of self-stigma and aimed to explore the experience of stigma of FSWs when accessing health care services. The predetermined coding categories for stigma from the literature were: experienced, anticipated, and internalized stigma. Besides, the classification of coping behaviors has been extensively studied [34, 35], it could be divided into two general categories: active coping and passive coping [34]. Therefore, a directed content analysis was adopted to analyze the interview data and field notes.

First, the transcriptions and field notes were read by two researchers independently without any attempts to conduct coding, to obtain an overall picture of the interview. A meaning unit is the smallest unit that contains aspects related to each other through their content and context (p.106) [36]. It could be words, phases, or sentences [36, 37]. The meaning units related to the participants’ experiences of stigma and coping strategies were identified and highlighted, which included simple and clear phrases and sentences, such as “bad attitudes”, “I feel ashamed of myself”, “I feel stressed on the way to the clinic”, etc. After that, the meaning units were coded with the predetermined coding categories if possible. The data that could not be coded in these categories were coded with other categories and themes by adopting the inductive approach. Examples of meaning units, summarized meaning units, sub-theme and theme are presented in Table 1. The two researchers discussed the resulting themes until they reached a consensus. Once no new concepts emerged from the data, the researchers re-examined the data and agreed upon a number of higher-order themes. Only after the themes were identified and confirmed were the quotations translated into English by the researchers for use in writing the report. The number at the end of each quote refers to the number assigned to the individual who was interviewed.

**Table 1:** Examples of meaning units, summarized meaning units, sub-theme and theme

Meaning unit	Summarized meaning unit	Sub-theme	Theme
*I visited a social hygiene clinic 3 years ago. The staff there probably suspected that I was a sex worker, because they were rude and spoke to me in harsh reprimanding voices. I felt humiliated. I definitely won’t go there again.*	Negative experience	Experienced stigma	Experience of stigma in the health care setting
*I was so scared and worried about being humiliated when I first sought help for STDs. I wore a mask and big sunglasses when I visited the clinic. As soon as I had completed my medical consultation, I ran away like “a rat scampering in the street.*	Worried about being humiliated	Anticipated stigma	
*It would be embarrassing to bump into acquaintances at the social hygiene clinics. I won’t seek help from public health services or social hygiene clinics.*	Being witnessed in the public health sector cause stress	Internalized stigma	
*I can be a housewife or a manager in a company. It is not necessary to tell health care workers the truth about my work when seeing a doctor. Even if I get HIV, it does not mean that I necessarily got it from my sex work. Everyone has a chance to become infected.*	Hide private information and to set boundaries with health care providers	Concealment of sex worker identity	Coping with the stigma of sex work
*I would go to the local NGOs for regular STDs tests. Because it is a sex worker-friendly organization, I feel safe and be respected there.*	Choose stigma-free alternative services	Avoidance of stigmatizing situations	
*I understand that not everyone accept sex workers. Therefore, I pay more attention to the disease treatment than the attitudes of the health care provider. Their perception of me would not affect my life*	Normalize others’ negative attitudes	Ignore the stigma	
*We could receive more comprehensive and necessary diagnostic tests and treatments if we disclose our sex work at the social hygiene clinic. Besides blood tests, they also offer a saliva test and a Pap smear test*	Analyze the risks and benefits	Selective disclosure of sex worker identity	
*Women engage in sex work for various reasons, many FSWs scarify their pride and dignity for their family. I need to pay for the rent, the tuition fee of my son, and the living expenses. Sex work is the only way for me to make a living and be a responsible mother. The health care providers could not imagine how difficult for a single mother living in Hong Kong. They should not judge me based on the sex work I do*	Use poverty as an excuse of sex work	Justification of sex work	
*My peers gave me great support. They encouraged me to have routine STDs tests and even accompanied me to the hospitals. This makes me feel less stressed when visiting the doctor.*	Handle stress with social support networks	Seek out social support	
*When I feel sad or unhappy, I will go out with friends and drown my sorrows with alcohol.*	Understand the complex needs	Multiple health care needs beside STDs	The call for non-judgmental holistic health care
*Only if health care providers have a good understanding of the sex industry and our work environment would they understand our occupational risks and be more sensitive to our multiple health care needs. They would also understand our fears, sorrows, and depression beyond those related to the contraction of STDs.*	Suggest for better health care services	Expand the scope of services	

In the qualitative study, member checking is considered as a crucial technique to ensure the accuracy, credibility and validity of the results [38, 39]. It was performed after the data analysis of the study. All the participants were invited to review the analyzed data and gave comments on the accuracy of the interpretation.

### Ethical considerations

The interviews were conducted with caution and with the guidance and support of NGOs. The well-being of FSWs was the central consideration of the study, which was considered as a driver of the design of the study and the reasons for the involvement of NGOs. Staff of NGOs helped to monitor the emotional reactions of FSWs during the interview and provided psychological support to the participants if needed. FSWs were also offered the number of a crisis hotline.

To avoid being identified, the participants were required to sign the informed consent by using pseudonyms. The participants had the rights to accept or refuse audio-recording. If they refuse to be recorded, handwritten notes would be taken by the researcher and staff of NGOs during the interview, and the participants would be asked to comment on the notes after the interview.

Each participant was offered HK$400 (US$1 USD ≈ HK$7.8) as compensation for their time and willingness to share their experiences in seeking health care. To ensure the confidentiality of the participants, they were not required to give their legal name. Participation in this study was voluntary, and numerical identifiers were used to protect the participant’s identity. FSWs’ decision on whether or not to participate in the study would not affect their current or future relationship with NGOs. They would be allowed to withdraw from the study at any time without penalty.

## Results

### Study population

Study participants were recruited from various settings with the assistance of NGOs. They included those who work in one-woman brothels (*n* = 18), massage parlors (*n* = 3), and those who are involved in compensated dating (*n* = 1). The participants were 30 to 59 years of age. The majority of FSWs were born in mainland China (*n* = 20), one was born in Hong Kong, and another in Vietnam. They had lived in Hong Kong from two to 20 years. Approximately half of them (*n* = 10) had received a primary school education, while the rest had received a middle school education (*n* = 12). All but one of them had had an unsuccessful marriage: two had separated from their spouse and 19 had divorced, with six of them having remarried. All but three participants had children, and 10 had had at least one induced abortion. The majority of them lived in government-subsidized public housing (*n* = 8) or in a rented apartment (*n* = 8), two lived in a private apartment, and four lived and worked in a rented one-woman brothel.

All the participants engaged in the sex industry for money and viewed sex work as a rational choice. The reasons were complex, and many factors were interrelated. The majority of the divorced women (*n* = 15) were confronted with great economic difficulties and viewed sex work as a means of survive, such as the responsibility of raising children, rent house, and live independently. Over half of the participants (*n* = 14) reported a lack of job opportunities. Nine of them complained about the low-paid labor work in the service industry, four reported limited job opportunities due to their health condition, and one could not speak the local language fluently. Besides, three participants needed to pay off the family debt. Only one sex workers worked for buying luxury goods.

The participants had been engaged in sex work for an average of 3.95 years (range 0.5–12 years), and were serving about 2 to 7 clients a day. Their monthly income ranged from HKD$4000 to HKD$100,000 (US$510–$12,800). Five of them had sources of income other than that derived from sex work.

In relation to self-protection in sex work, all except three of the participants used a condom consistently with their clients. However, two of them had had a condom slip off or removed by the client during intercourse, and 14 provided unprotected oral sex. All denied ever having engaged in anal sex.

### Health conditions and accessing health care services

The participants engaged in various types of health risk behaviors, such as smoking (*n* = 10), drinking alcohol (*n* = 5), gambling (*n* = 3), being shopaholics (*n* = 2), and using illicit drugs (*n* = 1).

The participants suffered from a range of diseases. STDs were the most frequently reported forms of disease, with urethritis being the most common (*n* = 10), followed by vaginitis (*n* = 5), chlamydia (*n* = 2), syphilis (*n* = 1), hepatitis B (*n* = 1), herpes (*n* = 1), and acute pelvic inflammatory disease (*n* = 1). The participants also suffered from chronic conditions, including hyperthyroidism (*n* = 2), hypoglycemia (*n* = 2), diabetes (*n* = 1), heart disease (*n* = 1), stomach ulcers (*n* = 1), endometrial polyps (*n* = 1), headache (*n* = 1), back pain (*n* = 1), and plantar fasciitis (*n* = 1).

All but two participants had ever sought health services in the past year (*n* = 19). The most common reasons for seeking help were for HIV/STDs tests or treatments (*n* = 15), followed by an annual health check-up (*n* = 6) and for the management of chronic diseases (*n* = 3).

The participants tended to seek health care from NGOs (*n* = 10), followed by social hygiene clinics (*n* = 7) and private doctors (*n =* 6). The participants had reservations about seeking health services from public hospitals in Hong Kong. Among those who sought such services, four did so when they returned to mainland China and one when she returned to Vietnam; only three were willing to do so in Hong Kong.

## Themes of the study

The interview data can be grouped into three themes: experience of stigma in the health care setting; coping with the stigma of sex work; and the call for non-judgmental holistic health care.

### Theme 1: Experience of stigma in the health care setting

The experience of stigma and discrimination among FSWs who accessed healthcare services varied. We found that 12 out of the 22 participants indicated that they did not experience discrimination from health care providers. Despite the long waiting time at the public health sectors, some commented favorably about the universal coverage of health care services in Hong Kong. In fact, the majority of the participants did not perceive the bad attitude of health care providers as a sign of stigma when seeking treatment. Instead, they perceived all patients were treated equally, or treated with equally bad attitudes.

By contrast, some FSWs had experienced stigmatized attitudes from health care providers when they sought treatment for their STDs. The participants believed that the stereotypes held by health care providers were that women who contracted STDs were sex workers and fallen women. They may experience, anticipate, or internalize stigma when accessing health care services.

### Experienced stigma

The participants complained that health care providers, especially those from the public health sector, hold negative and discriminatory attitudes towards them. A participant described her unfortunate experience at a social hygiene clinic.

*I visited a social hygiene clinic 3 years ago. The staff there probably suspected that I was a sex worker, because they were rude and spoke to me in harsh reprimanding voices. I felt humiliated. I definitely won’t go there again. (#5)*

### Anticipated stigma

FSWs believed health care providers held prejudiced attitudes toward sex work and STDs, and would judge them as sinful and diseased. Being worried about and anticipating or having experienced disdain from health care providers, FSWs accentuated their self-stigma when they were forced to access health care services for STDs:

*I was so scared and worried about being humiliated when I first sought help for STDs. I wore a mask and big sunglasses when I visited the clinic. As soon as I had completed my medical consultation, I ran away like “a rat scampering in the street.” (#12)*

### Internalized stigma

The experienced stigma and the anticipated stigma could lead FSWs to internalize the prejudice, manifesting in shame, fear, and low self-esteem. The majority of FSWs felt ashamed of their occupation. They feared that their identity as a sex worker might be revealed in the process of visiting STDs clinics, and were worried about the consequence of being identified as a sex worker, such as gossip and discriminated by health care providers.

*I felt ashamed of myself when I visited the social hygiene clinic. A good woman does not need to have the STDs examination. Health care providers must associate me with a sex worker and a dirty woman. They must look down on me. (#5)*

FSWs believed the general public, including their “sex customers,” held prejudiced attitudes toward sex work and STDs. They would feel ashamed if they were witnessed visiting the public STDs clinic.

*It would be embarrassing to bump into acquaintances at the social hygiene clinics. I won’t seek help from public health services or social hygiene clinics. (#11)*

In summary, FSWs acknowledged that the sex trade and STDs were socially despised. They had experienced or anticipated stigma and discrimination in the health care setting. The perceived lack of public acceptance when they sought help at health services clinics for STDs led to a feeling of stress, fear, and shame.

### Theme 2. Coping with the stigma of sex work

The participants adopted various strategies to cope with the stigma associated with sex work and STDs in the health care setting. Those who accepted the social stigma of sex work may adopt passive coping strategies, including the concealment of sex worker identity, avoidance of stigmatizing situations, and ignore the stigma. FSWs who resisted the social stigma of sex work may adopt active coping strategies, including selective disclosure of sex worker identity, justification of sex work, and seek out social support. Below is a description of the coping strategies.

### Passive coping

#### Concealment of sex worker identity

The majority of the participants worried that if they disclosed their sex work they would be inviting moral judgments from health care providers and gossip about their identity, leading to shame and embarrassment as well as possibly impacting the care that they would receive. Thus, the majority would attempt to protect their privacy when seeking health care services. For example:

*I will lose face if I disclose my sex worker identity to the health care provider. It is an untold secret. (#1)*

Some would lie about their work. For example, one participant commented:

*I can be a housewife or a manager in a company. It is not necessary to tell health care workers the truth about my work when seeing a doctor. Even if I get HIV, it does not mean that I necessarily got it from my sex work. Everyone has a chance to become infected. (#13)*

Sex work is a taboo in the health care setting, and most of FSWs were aware that health care providers in Hong Kong are not allowed to directly ask them this sensitive question. One FSW described how a doctor asked her about her sexual activities:

*Once I went to a clinic for STDs or gynaecological diseases, and I could tell that the doctor there suspected me of engaging in sex work, but he knew that it would be offensive if he asked directly. Instead, he asked me whether or not I use a condom with my partner and whether or not I have a job. (#19)*

#### Avoidance of stigmatizing situations

Some FSWs believed health care providers, especially those from public health sectors, held prejudiced attitudes toward sex work and STDs. To avoid situations that may result in stigma and discrimination, many FSWs preferred to use clinics operated by NGOs, where they could receive both informational and emotional support and enjoyed free condoms and sexual and reproductive health care services. They did not have to worry about disclosing their sex work to NGOs since the service was anonymous.

*I would go to the local NGOs for regular STDs tests. Because it is a sex worker-friendly organization, I feel safe and be respected there. (#4)*

To avoid being identified as a sex worker, some FSWs would visit a hospital of the neighbouring city or their hometown in mainland China. Moreover, they commented that the service in mainland China was more convenient and comprehensive, and they did not to feel embarrassed since they could avoid talking about STDs.

*If I want to get sexual health check-up, I can go to the department of obstetrics and gynaecological of a hospital instead of STDs clinics. No one will associate me with a sex worker there. Also, I could have a full body check-up without mentioning STDs tests. (#7)*

#### Ignore the stigma

Ignore the attitudes of health care providers was considered as an important strategy to buffer against the stress and fear when accessing health care services. Many participants built resilience and had learnt to ignore others’ perception. As one participant explained:

*I understand that not everyone accept sex workers. Therefore, I pay more attention to the disease treatment than the attitudes of the health care provider. Their perception of me would not affect my life. (#3)*

### Active coping

#### Selective disclosure of sex worker identity

FSWs would weigh the risks and benefits of revealing their identity. Sometimes, the perceived benefits of revealing the truth to receive appropriate and timely diagnostic tests and medical treatment might trigger the decision to make the disclosure.

*We could receive more comprehensive and necessary diagnostic tests and treatments if we disclose our sex work at the social hygiene clinic. Besides blood tests, they also offer a saliva test and a Pap smear test. (#12)*

*When a serious illness such as HIV is suspected, it is better for us to disclose our sex work because it is important information that will help the doctors and nurses to decide on the diagnostic tests and treatment plan. Only if we tell the truth can we get prompt treatment. (#9)*

The participants also commented on the supportive health care environment that empowered them to be open. The participants were confident about the maintenance of confidentiality in the public and private health sectors, and therefore did not see the need to conceal their identity from health care providers.

There was a gradual change in FSWs’ attitudes toward STDs services. Several FSWs admitted that they felt embarrassed and ashamed to have STDs tests when they entered into the sex industry, and were reluctant to reveal their identity to health care providers at the social hygiene clinic. Only after they became acquainted with them and had established mutual trust were they able to disclose their sex work. They observed that the attitudes of health care providers did not change after they disclosed their secret.

*The attitude of health care providers in the social hygiene clinic did not change after I disclosed my sex work. The nurse was gentle when she was examining me. She also spoke softly, telling me to “Relax, relax!” (#15)*

#### Justification of sex work

In most circumstances, FSWs resisted the stereotype that sex work were immoral or deviant. They tended to justify sex work as a personal and rational choice, and were not ashamed of engaging in it. They felt that, as divorced women, single mothers, and lacking in education and other skills, they had limited job opportunities and choices. They confided that sex work offers economic benefits, flexible work hours, and allows them to provide their family with the necessities of life. As an FSW commented:

*Women engage in sex work for various reasons, many FSWs scarify their pride and dignity for their family. I need to pay for the rent, the tuition fee of my son, and the living expenses. Sex work is the only way for me to make a living and be a responsible mother. Health care providers should not judge me based on the sex work I do. (#20)*

Some even suggested that their work could reduce the incidence of rape for the public good. These FSWs justified their sex work as labor they undertook to support their family and felt empowered to disclose their identity to health care providers. For example:

*The attitudes of health care providers won’t upset me. I have no other choice, and I am proud that I can make a living for myself. I also think that sex workers have helped to reduce the incidence of rape and the crime rate. (#16)*

The participants also emphasized that in Hong Kong, commercial sexual services between two adults was not illegal, and being an FSW was not illegal as long as one serves in a one-woman brothel and was legally resident in Hong Kong. The participants were free from the fear of being arrested even if they disclosed their work.

#### Seek out social support

Social support played vital role in reducing the fears and stress of FSWs. Many participants were accompanied by peers or staff of NGOs during their visit to doctors. The emotional support and the resilience of peers who against the stigma of sex work helped to reduce their psychological stress. A FSW commented:

*My peers gave me great support. They encouraged me to have routine STDs tests and even accompanied me to the hospitals. This makes me feel less stressed when visiting the doctor. (#12)*

In summary, the interviewed FSWs adopted various strategies to combat stigma in the health care setting. The majority of FSWs chose to hide their identity due to the fear of stigma. Those who were able to disclose their identity were empowered by their open-minded attitude towards sex work, the perceived benefits of disclosing their identity, and the perception of a supportive health care environment. Sometimes, FSWs would ignore the attitudes of health care providers or seek help from the place where they felt safe and friendly. Some FSWs tended to justify sex work and emphasize their contribution to their family and the society. Moreover, the social support they received allowed them to deal with the stress and fear when accessing health care services.

### Theme 3: the call for non-judgmental holistic health care

The majority of the participants believed they would more readily access health care services if the health care team had a good understanding of the sex industry, recognized them as people, and treated them holistically with dignity. Besides sexual health, they desired comprehensive and holistic health care which could take into consideration of their multiple health care needs, such as mental disorders, diabetes, hypoglycemia, insomnia, plantar fasciitis, problem gambling, and other addictions.

*“Our comprehensive health care needs should be addressed. For example, my heel is killing me, and I could not walk a long distance. However, I have no idea where to seek help. I wish someone could help me with these problems other than STDs.” (#15)*

#### Multiple health care needs beside STDs

Due to life difficulties/traumas and the stigma associated with sex work and STDs, many participants had developed mental health problems, such as severe stress, anxiety, insomnia, and depression, and some had even attempted suicide. Several participants engaged in various types of addiction to cope with the difficulties of their life and with emotional disorders, including chain smoking, drinking alcohol, binge drinking, shopping, taking drugs, and gambling. All except one did not seek mental health care services. The one person who had visited a mental health care provider was merely told “not to think too much.” She then drank a great deal of alcohol to deal with her depression and sadness.

*When I feel sad or unhappy, I will go out with friends and drown my sorrows with alcohol. (#17)*

Another FSW who suffered from a gambling addiction described her emotional despair:

*I am a gambler! That way I can free myself from thinking of my troubles. But once I lost a huge amount of money in a casino. I hated myself so much and attempted to commit suicide with a knife. Eventually, I called the police for help. (#15)*

#### Expand the scope of services

FSWs with multiple health care needs made a strong call for the provision of non-judgmental holistic care. Some participants highlighted the needs for health care providers to understand the sex industry and their occupational health and safety.

*Only if health care providers have a good understanding of the sex industry and our work environment would they understand our occupational risks and be more sensitive to our multiple health care needs. They would also understand our fears, sorrows, and depression beyond those related to the contraction of STDs. (#14)*

Further, some FSWs spoke very favorably of the free sexual health services provided by the social hygiene clinic and NGOs, especially the non-judgmental care and outreach services provided by NGOs. However, they also highlighted that services provided by these organizations were not comprehensive enough, and they made a series of recommendation for the expansion of health care services. For example:

*Sometimes, I feel depressed. But I never seek help from a health professional because I can neither afford the years-long waiting time at the public health sector nor afford the expenses in the private health sector. Since we have regular STDs screening tests at NGOs or the social hygiene clinic, it would be great if they could offer more supportive services, like psychological counselling or referral to mental health treatment. (#15)*

This theme revealed that besides STDs, FSWs had multiple health care needs. They were also at risk of developing mental disorders and addictions as a result of social stigma and life difficulties. STD clinics or NGOs should take a holistic approach that considers multiple health care needs when caring for FSWs.

## Discussion

The study aimed to investigate the experience of stigma in the health care setting and stigma coping strategies among FSWs in Hong Kong. Generally speaking, stigma was not viewed as a concern for some FSWs unless they sought for STDs services from the public STDs clinic. The participants believed that the stereotypes held by health care providers were that women who contracted STDs were sex workers. The finding of this study is consistent with literature showing that, for FSWs, stigma is an important issue when accessing HIV/STDs health care services [9, 40, 41]. Despite the available, accessible, and affordable public health care services in Hong Kong [15], being a sex worker or having STDs is not socially acceptable and sometimes a significant concern for FSWs when seeking help from health care providers.

The results revealed the flexibility of FSWs in responding to the stigma of sex work and the associated STDs in the health care setting. Their choice of stigma coping strategies varied as a result of the self-perception, the perception of the occupation, the perception of STDs and the severity of the disease, the perceived risks and benefits, the complex interactions with health care providers, and the availability of social supports. This finding provides insights into FSWs’ internal dilemma, on making a decision whether or not to disclose their identity. Consistent with reports in the literature that FSWs rarely reveal their sex worker identity when seeking professional help [11, 42], the paradox of coming out as a “sex worker” was considered as most challenging for the majority of FSWs in this study. However, holding back one’s feelings and emotions could lead to stress and subsequent physical health problems [43]. The burden of internalized stigma and perceived stigma from the public and health professionals could lead to a vicious cycle of internalized stigma, poor self-esteem, and illness.

It was quite encouraging to notice that a few participants were empowered to open themselves up to face the stigma in society. The perceived seriousness of their health condition and the potential benefits of disclosing their identity may cause them to feel a pressing need to respond to their health problems and prompt them to disclose their private information to health care providers. Such disclosure often invited more support from health care providers, such as comprehensive and timely diagnostic tests and treatments, empathetic, respectful, and non-judgmental care, and free resources and services. Meanwhile, findings from this study further indicated that the support in the health care setting facilitated FSWs’ access of health care services and the disclosure of private information. Similar findings have been reported in other countries that the disclosure of sex work could lead to increased social support and vice versa [44]. Thus, it is crucial to raise the awareness of health care providers that their support could help to end the vicious cycle of stigma and illness among FSWs. The provision of a friendly environment offering non-judgmental health services could help to mitigate the stigma felt by FSWs and encourage them to access the services. The sexual and reproductive health service provided by NGOs was considered to be friendly and sensitive, which facilitates the provision of better services and bolsters the service uptake rate.

In addition, results from the study highlight the need to address multiple health needs of FSWs. Besides STDs, FSWs also need support for other conditions, such as mental illness and addictions. However, they are facing barriers to access specialty care which could have a significant impact on their health. As many of FSWs have regular STDs check-ups, health care providers of STDs clinics and NGOs should be sensitive to the needs of FSWs and offer referral to those who need specialty care. Besides, it is suggested that a multidisciplinary team may be considered to integrate mental health services and addiction with STDs services.

## Implications

The stigma of sex work and the associated STDs may influence the experience of health care services among FSWs, especially the experience of STDs services. In order to improve FSWs’ experience of health care, interventions programmes could be conducted at different levels.

At the individual level, interventions are needed to reduce FSWs’ internalized stigma According to the social identity theory [45], identity management strategies may help members of the stigmatized group cope with stigma, restore their positive social identity, and improve the self-esteem. Regarding various coping strategies FSWs may adopt, researchers are suggested to take into the perspectives of FSWs and find the fit identity management strategy with which FSWs feel comfortable. Also, self-stigma reduction interventions among people with other stigmatized conditions could be used as a reference to develop the intervention to reduce the self-stigma among the sex workers, such as psychoeducation, cognitive restructuring, and narrative intervention [46, 47].

At the instructional level, it is crucial to raise health care providers’ awareness of the stigma or subconscious bias toward FSWs. Health care providers and students in the health care professions should participate in sensitivity-training programs. These could focus on increasing their awareness and understanding of the sex industry, increasing their knowledge about the multiple health risks and health care needs of FSWs, and improving their history-taking skills and their ability to encourage FSWs to disclose their health concerns, and instructing them on how to promote a friendly and non-judgmental medical environment. The intergroup contact theory suggested that the intergroup contact under the conditions of equal status, common goals, intergroup cooperation, and institutional support could reduce the bias and improve understanding [48]. This approach may be used to reduce stigmatized attitudes towards sex workers among health care providers.

Furthermore, communications with FSWs should not be based on the assumption that they were merely vulnerable to contracting HIV/STDs, since this study also revealed that FSWs face other work-related risks beyond STDs, such as mental illness, addictions, and other chronic diseases. Health care providers need to conduct a comprehensive assessment of all clients, using patient-centered care principles.

At the societal level, community empowerment may be used to promote a respectful environment for FSWs. The Sonagachi Project in India achieved success in reducing the social stigma toward sex workers as well as empowering sex workers [49]. It promoted the human rights, provided condoms and material resources, and created a sense of collective identity among FSWs. The local NGOs in Hong Kong could play an essential role in promoting the recognition and decriminalization of sex work, which, in turn, empower FSWs when accessing health care services. Furthermore, open discussions on the best legal framework for dealing with prostitution and protecting the human rights of prostitutes should be encouraged.

## Limitations of the study

The study was conducted among a subgroup of FSWs in Hong Kong (those operating out of one-woman brothels). The findings of this study may not be applicable to other groups of FSWs. A further study should be conducted of other subgroups of FSWs, such as adolescent FSWs, sex trafficked women, migrant FSWs, or FSWs based in other venues.

Second, due to the highly sensitive nature of the topic, the possibility exists that FSWs gave socially desirable responses when describing their health, sexual activities, and health behaviors towards the utilization of health care services.

## Conclusion

Although stigma does not affect all FSWs when accessing health care services in the study, it remains an important issue for a significant proportion of FSWs when they seek timely professional help, fully disclose their secret of being involved in sex work, and receive comprehensive health care services. Thus, stigma is still an important aspect to address. The study also contributes to the existing literature on various coping strategies that FSWs adopted in dealing with stigma in the health care setting. Findings from the study also highlight the need for understanding and addressing multiple healthcare needs of FSWs, and NGOs and the social hygiene clinic may consider expand its services to other health concerns beyond STDs. Moreover, it contributes to increasing awareness of, and respect for, the health care needs and human rights of FSWs among health care professionals and students in the health care professions.

## Supplementary information

**Additional file 1.** Qualitative interview guideline.

## Data Availability

The datasets during and / or analysed during the current study available from the corresponding author on reasonable request.

## References

[1] Erving G (1963). Stigma: notes on a spoiled identity.

[2] Durisin EM, Van der Meulen E, Bruckert C. Red Light Labour: Sex Work Regulation, Agency, and Resistance. Vancouver: UBC Press; 2018.

[3] Poutanen MA (2015). Beyond brutal passions: prostitution in early nineteenth-century.

[4] Rekart ML (2005). Sex-work harm reduction. Lancet.

[5] Corrigan PW, Watson AC (2002). Understanding the impact of stigma on people with mental illness. World Psychiatry.

[6] Corrigan PW, Lam C. Challenging the structural discrimination of psychiatric disabilities: lessons learned from the American disability community. Rehabil Educ. 2007;21(1):53-8.

[7] Ma PH, Chan ZC, Loke AY (2017). The socio-ecological model approach to understanding barriers and facilitators to the accessing of health services by sex workers: a systematic review. AIDS Behav.

[8] Rahmati-Najarkolaei F, Niknami S, Aminshokravi F, Bazargan M, Ahmadi F, Hadjizadeh E (2010). Experiences of stigma in healthcare settings among adults living with HIV in the Islamic Republic of Iran. J Int AIDS Soc.

[9] Beattie TS, Bhattacharjee P, Suresh M, Isac S, Ramesh B, Moses S (2012). Personal, interpersonal and structural challenges to accessing HIV testing, treatment and care services among female sex workers, men who have sex with men and transgenders in Karnataka state, South India. J Epidemiol Community Health.

[10] Stangl AL, Earnshaw VA, Logie CH, van Brakel W, Simbayi LC, Barré I (2019). The health stigma and discrimination framework: a global, crosscutting framework to inform research, intervention development, and policy on health-related stigmas. BMC Med.

[11] Urada LA, Simmons J (2014). Social and structural constraints on disclosure and informed consent for HIV survey research involving female sex workers and their bar managers in the Philippines. J Empir Res Hum Res Ethics.

[12] Hunt J, Bristowe K, Chidyamatare S, Harding R (2017). ‘They will be afraid to touch you’: LGBTI people and sex workers’ experiences of accessing healthcare in Zimbabwe—an in-depth qualitative study. BMJ Global Health.

[13] Census and Statistics Department. The Government of the Hong Kong Special Administrative Region. Available from: https://www.censtatd.gov.hk/hkstat/sub/so20.jsp. Accessed 11 Nov 2019.

[14] Department of Health. Vision, mission and core values. The Government of the Hong Kong Special Administrative Region Available from: https://www.dh.gov.hk/english/aboutus/aboutus_mv/aboutus_mv.html. Accessed 11 Nov 2019.

[15] Kong X, Yang Y, Gao J, Guan J, Liu Y, Wang R (2015). Overview of the health care system in Hong Kong and its referential significance to mainland China. J Chin Med Assoc.

[16] Ziteng (2001). The sex trade industry in Hong Kong: a call for activism and transformation.

[17] Emerton R, Laidler KJ, Petersen CJ (2007). Trafficking of mainland Chinese women into Hong Kong’s sex industry: problems of identification and response. Asia Pac J Hum Rts & L.

[18] Cheung N. Accounting for and managing risk in sex work: a study of female sex workers in Hong Kong (Doctoral dissertation, Royal Holloway, University of London); 2012.

[19] Hong Kong Crimes Ordinance. Cap. 200 S147 Soliciting for an immoral purpose. (1990). Available from: https://www.elegislation.gov.hk/hk/cap200. Accessed 11 Nov 2019.

[20] Wong WC, Gray SA, Ling DC, Holroyd EA (2006). Patterns of health care utilization and health behaviors among street sex workers in Hong Kong. Health Policy.

[21] Lau JT, Tsui H, Ho SP, Wong E, Yang X (2010). Prevalence of psychological problems and relationships with condom use and HIV prevention behaviors among Chinese female sex workers in Hong Kong. AIDS Care.

[22] Lau JT, Mui LW, Tsui H, Wong E, Ho SP (2007). Prevalence of induced abortion and associated factors among Chinese female sex workers in Hong Kong. J Sex Marital Ther.

[23] Wong HT, Lee KC, Chan DP (2015). Community-based sexually transmitted infection screening and increased detection of pharyngeal and urogenital Chlamydia trachomatis and Neisseria gonorrhoeae infections in female sex workers in Hong Kong. Sex Transm Dis.

[24] Center for Health Protection DoH (2016). HARiS - HIV and AIDS response indicator survey 2015 for female sex worker.

[25] Corrigan PW, Watson AC (2002). The paradox of self-stigma and mental illness. Clin Psychol Sci Pract.

[26] Watson AC, River LP (2005). A social-cognitive model of personal responses to stigma.

[27] Al-Busaidi ZQ (2008). Qualitative research and its uses in health care. Sultan Qaboos Univ Med J.

[28] Weiss MG (2001). Cultural epidemiology: an introduction and overview. Anthropol Med.

[29] Kaplowitz MD, Hoehn JP (2001). Do focus groups and individual interviews reveal the same information for natural resource valuation?. Ecol Econ.

[30] Faugier J, Sargeant M (1997). Sampling hard to reach populations. J Adv Nurs.

[31] Mishler EG. Research interviewing. Context and narrative. Cambridge: Harvard University Press;1986.

[32] Francis JJ, Johnston M, Robertson C, Glidewell L, Entwistle V, Eccles MP (2010). What is an adequate sample size? Operationalising data saturation for theory-based interview studies. Psychol Health.

[33] Hsieh H-F, Shannon SE (2005). Three approaches to qualitative content analysis. Qual Health Res.

[34] Brown GK, Nicassio PM (1987). Development of a questionnaire for the assessment of active and passive coping strategies in chronic pain patients. PAIN®.

[35] Folkman S, Lazarus RS. An analysis of coping in a middle-aged community sample. J Health Soc Behav. 1980:219–39. 7410799

[36] Graneheim UH, Lundman B (2004). Qualitative content analysis in nursing research: concepts, procedures and measures to achieve trustworthiness. Nurse Educ Today.

[37] Mostyn B (1985). The content analysis of qualitative research data, a dynamic approach. The research interview, users and approaches.

[38] Lincoln YS (2007). Naturalistic inquiry. The Blackwell encyclopedia of sociology.

[39] Creswell JW, Poth CN. Qualitative inquiry and research design: choosing among five approaches: Sage Publications; 2016.

[40] Scorgie F, Nakato D, Harper E, Richter M, Maseko S, Nare P (2013). ‘We are despised in the hospitals’: sex workers’ experiences of accessing health care in four African countries. Cult Health Sex.

[41] Mtetwa S, Busza J, Chidiya S, Mungofa S, Cowan F (2013). “You are wasting our drugs”: health service barriers to HIV treatment for sex workers in Zimbabwe. BMC Public Health.

[42] Ndung'u CN. Barriers faced by female sex workers in seeking healthcare at public health facilities in mlolongo ward, athi-river sub-county (Doctoral dissertation, University of Nairobi); 2016.

[43] Paxton S (2002). The paradox of public HIV disclosure. AIDS Care.

[44] Benoit C, Smith M, Jansson M, Magnus S, Maurice R, Flagg J, et al. Canadian sex workers weigh the costs and benefits of disclosing their occupational status to health providers. Sex Res Soc Policy. 2018;16(3):329–41. 10.1007/s13178-018-0339-8 PMC6669194 31423291

[45] Tajfel H, Turner JC, Austin WG, Worchel S (1979). An integrative theory of intergroup conflict. Organizational identity: a reader.

[46] Yanos PT, Lucksted A, Drapalski AL, Roe D, Lysaker P (2015). Interventions targeting mental health self-stigma: a review and comparison. Psychiatr Rehabil J.

[47] Ma PH, Chan ZC, Loke AY. Self-stigma reduction interventions for people living with HIV/AIDS and their families: a systematic review. AIDS Behav. 2018:1–35. 10.1007/s10461-018-2304-1 30298241

[48] Allport GW, Clark K, Pettigrew T (1954). The nature of prejudice.

[49] Gangopadhyay DN, Chanda M, Sarkar K, Niyogi SK, Chakraborty S, Saha MK (2005). Evaluation of sexually transmitted diseases/human immunodeficiency virus intervention programs for sex workers in Calcutta, India. Sex Transm Dis.

