# Supplementary material for: A qualitative study into female sex workers’ experience of stigma in the health care setting in Hong Kong
Source: Int J Equity Health. 2019 Nov 14;18:175. doi: 10.1186/s12939-019-1084-1 (PMC6857210; doi:10.1186/s12939-019-1084-1)
Supplement: Supplementary file 1 — Additional file 1. Qualitative interview guideline. [file 12939_2019_1084_MOESM1_ESM.docx]

**Qualitative interview guideline**

1. Can we start by sharing with me what a typical day at work is like for you and what kind of health problems you encounter?
2. Can you recall your health care experiences?

Prompting questions: Can you describe the symptoms of the illness? How about the health care settings? How did the health care professionals treat you? What made you think…? Can you tell me more about…? What did you do about…?

Target question(s) to get at the “core” of your concern about health care needs and health care services.

1. How did you manage your concern when the health care professionals suspect you were a sex worker?

Prompting questions: What goes through your mind as you try to seek health care services? Can you tell me more about the concerns or discomfort you feel? What did you do when....?

1. How did you feel after seeking health care services?

Prompting questions: How did you come to feel that way? What makes it difficult to disclose your information? How do you feel about being asked directly/suspected about your sex work? Can you tell me more about...?

1. How do you feel about the idea of workshops between sex workers and health care professionals to increase mutual understanding?

Is there anything you’d like to add?

Thank you!
